# Insulin Resistance and Its Association with Metabolic Syndrome in Korean Children

**DOI:** 10.1155/2017/8728017

**Published:** 2017-12-31

**Authors:** Jinkyung Cho, Haeryun Hong, Soohyun Park, Shinuk Kim, Hyunsik Kang

**Affiliations:** ^1^College of Sport Science, Sungkyunkwan University, Suwon, Republic of Korea; ^2^Department of Sports Science, Korea Institute of Sport Science, Seoul, Republic of Korea; ^3^Department of Civil Engineering, Sangmyung University, Cheonan, Republic of Korea

## Abstract

**Background:**

This study investigated the association between insulin resistance (IR) and metabolic syndrome (MetS) in children.

**Methods:**

A cross-sectional study involving 1036 healthy children aged between 7 and 13 years was conducted. Homeostasis model assessment of insulin resistance (HOMA-IR) was calculated as an index of IR. Participants were classified according to the HOMA-IR quartiles.

**Results:**

Incremental, linear trends were found in age (*p* < 0.001), body mass index (BMI) (*p* < 0.001), body fat (*p* < 0.001), waist circumference (*p* < 0.001), resting blood pressures (BP) (*p* < 0.001), triglycerides (TG) (*p* < 0.001), total cholesterol (TC) (*p* < 0.001), high density lipoprotein-cholesterol (HDL-C) (*p* < 0.001), FBG (*p* < 0.001), and insulin (<0.001) according to incremental HOMA-IR categories (from the 1st to 4th quartile). Compared with children in the 1st HOMA-IR quartile, children in the 4th HOMA-IR quartile had significantly higher odd ratios (ORs) of abnormalities in systolic (*p* = 0.051) and diastolic BP (*p* = 0.005), FBG (*p* < 0.001), TG (*p* < 0.001), TC (*p* = 0.016), and HDL-C (*p* = 0.006) even after adjustments for age, gender, BMI, and body fat percentage. Children in the 3rd HOMA-IR quartile had significant abnormalities in FBG (*p* < 0.001), TG (*p* = 0.001), and HDL-C (*p* = 0.010) even after adjustments for the covariates.

**Conclusion:**

The current findings suggest that IR is significantly associated with the clustering of MetS risk factors in children in Korea.

## 1. Introduction

Insulin resistance (IR) is defined as a defective metabolic response of insulin to stimulate glucose uptake into skeletal muscle and adipose tissue and/or to suppress hepatic gluconeogenesis and glucose release into circulation [1]. The metabolic response of IR and subsequent hyperinsulinemia is attributed to the development of serious health consequences such as overweight, hypertension, hyperlipidemia, cardiovascular disease, and type-2 diabetes (T2D) [1]. Previously, IR was described as a metabolic condition exclusive to adults. However, studies indicate that IR is also present in children and adolescents. The occurrence and abnormal components of IR are rising in children [2]. Adverse health effects of IR have also been documented in the pediatric population [3]. Metabolic syndrome (MetS) represents a clustering of metabolic risk factors including central obesity, hyperinsulinemia, hypertension, and dyslipidemia. Although the exact etiology of MetS is uncertain, IR is considered a common mechanism underlying derangements associated with the syndrome [4].

Koreans are prone to the development of IR and T2D due to impairment of early-phase insulin secretion capacity [5]. In addition, lack of physical activity (PA) and excessive caloric intake are becoming more prevalent in Korea [6]. Industrialization and the rapid growth of the technology sector have led to further decreases in PA including school- and home-based exercises and increases in sedentary lifestyles due to TV, personal computers, and internets [6]. For example, associations between IR and MetS risk factors have been reported in Korean patients with hyperlipidemia [7] and the middle-aged Korean offspring of hypertensive patients [8]. In 2002, the Korean National Health Examination and Nutrition Survey reported that, compared with the data from 1998, children have experienced significant increases in body weight and body mass index, along with other risk factors for developing IR [9]. A significant association between IR and MetS has also been reported in previous studies involving children and adolescents in Korea [10].

The natural history of IR as a common trigger of metabolic disorders associated with MetS in adulthood begins in childhood due to the interaction of genetic and environmental factors. Consequently, examining IR and its relationship to MetS in childhood plays an important role in identifying children with an increased risk of developing cardiovascular diseases such as T2D. However, there is limited data available regarding IR and its association with MetS in childhood, especially in Korea. This cross-sectional study aimed to explore the associations between IR and abnormalities in each of the MetS components in apparently healthy children aged 7–13 years in Korea.

## 2. Methods

### 2.1. Subjects

This study was conducted as part of an annual health and physical examination of elementary school students from April to June 2012. Initially, a total of 1440 children (670 boys and 770 girls) aged 7–13 years were voluntarily recruited from an elementary school located in the city of Ilsan, Republic of Korea. The children and their parents were then invited to an orientation at school and provided with a detailed explanation regarding the study.

Our institutional research ethics committee, in accordance with the Declaration of Helsinki of the World Medical Association, reviewed and approved the current study, and we obtained signed informed consent from both children and parents who agreed to participate. Among the initial 1440 children, a total of 404 children (180 boys and 224 girls) did not have blood sample, fasting blood sample, or body composition data due to various personal reasons. Consequently, the final sample consisted of 1036 children (488 boys and 548 girls) for data analyses.

### 2.2. Anthropometrics and Blood Pressure Measurements

Height and body mass were measured to the nearest 0.5 cm and 0.1 kg, respectively, using a scale with an attached stadiometer (Jenix, Seoul, South Korea). Body mass index (BMI) was calculated by the weight (kg) divided by the height squared (m^2^). Waist circumference (WC) was measured to the nearest 0.1 cm at the end of a normal expiration by placing a cloth tape at the midpoint between the lowest rib and the uppermost lateral border of the right iliac crest. Percentage of body fat was assessed using the X-scan bioelectrical body composition analyzer (Jawon Medical Co., Kyungsan, South Korea). Resting blood pressure (BP) was measured with an automated BP instrument (Jawon Medical Co., Kyungsan, South Korea) after the subject rested for 5 min in a seated position, with the arm at heart level and resting on the armrest of a chair. Two BP readings were recorded, and the average value was used in the analyses.

### 2.3. Blood Sample

Blood samples were drawn with the subjects in the supine position, following an overnight 10-hr fast. The fasting state was verbally confirmed by the subject before blood sampling. Fasting blood glucose (FBG), total cholesterol (TC), triglycerides (TG), and high density lipoprotein-cholesterol (HDL-C) levels were measured in duplicate using the Ektachem DT-60 II analyzer (Johnson & Johnson Clinical Diagnostics, Inc., Rochester, NY, USA). Fasting insulin was also measured in duplicate using a commercially available enzyme-linked immunosorbent assay (ELISA) kit (ALPCO Diagnostics, Salem, NH, USA). The index of insulin resistance was assessed using the homoeostasis model of assessment for insulin resistance (HOMA-IR), as HOMA-IR = [fasting insulin (uU/ml) × FBG (mM)]/22.5. The coefficients of variation (CVs) for intra- and interassays were 4.3% and 6.8%, respectively, for insulin.

### 2.4. Definition of Insulin Resistance and Metabolic Syndrome

IR was determined by the homeostasis model assessment of insulin resistance (HOMA-IR): [(FBG (mg/dL) × fasting insulin (*μ*U/mL))/405]. MetS was defined according to the metabolic syndrome definition of the International Diabetes mellitus Federation (IDF), except for the BP criteria as described below. Central obesity was defined as waist circumference > 90th percentile based on Korean waist circumference reference data [11]. Two or more of the following clinical features were additionally required: FBG concentration ≥ 100 mg/dL (5.5 mmol/L), fasting TG concentrations ≥ 150 mg/dL, systolic and/or diastolic BP of ≥90th percentile based on the child's age and gender, and HDL-C concentration < 40 mg/dL for both boys and girls.

### 2.5. Statistical Analyses

The distribution of variables was checked using univariate plots (e.g., histograms) and statistics (e.g., skewness and kurtosis). Outcomes of anthropometrics and metabolic risk factors are presented as means and standard deviations. Gender differences for the measured variables were examined using the Student's *t*-test. The children were classified into quartiles based on the distribution of means in HOMA-IR, as suggested previously [12]. Then we used general linear models to test linear trends for each mean of the measured metabolic risk factors across HOMA-IR quartiles by entering the HOMA-IR variable into the model as an ordinal term. Analyses were conducted separately for each of the metabolic risk factors. Analyses were performed with and without selected covariables (age and sex) that might influence the relationships between HOMA-IR and the metabolic risk factors. Finally, logistic regression analyses were used to calculate the odds ratio (OR) and 95% confidence interval (95% CI) of the MS components according to the HOMA-IR quartiles before and after adjustments for age, sex, BMI, and body fat percentage. All statistical tests were performed with SPSS-PC (version 21.0) with a *p* value of ≤0.05 as statistically significant.

## 3. Results

Table 1 shows the mean age, body fat, resting blood pressure, and metabolic profiles of children who participated in the study. Girls had significantly higher body fat percentage (*p* = 0.007) and FBG (*p* = 0.005) values than boys. In addition, girls tended to have a higher WC value (*p* = 0.091) than boys, while there were no significant differences between boys and girls in any of the other measured parameters. With respect to MetS, 48.4% and 31.7% of the children had 0 and 1 of the MetS components, respectively, while 14.3% and 5.7% had two and three or more of the components, respectively. Overall, girls (6.8%) tended to have a higher prevalence of MetS (*p* = 0.051) than boys (4.5%).

Anthropometric data and metabolic risk profiles according to HOMA-IR categories are shown in Table 2. There were significant positive, linear trends in the mean age (*p* < 0.001), BMI (*p* < 0.001), body fat (*p* < 0.001), WC (*p* < 0.001), SBP (*p* < 0.001), DBP (*p* < 0.001), TG (*p* < 0.001), TC (*p* < 0.001), FBG (*p* < 0.001), and insulin (<0.001) according to the incremental HOMA-IR categories (from the 1st to 4th quartile). In addition, there was a significant negative, linear trend in HDL-C (*p* < 0.001) according to the incremental HOMA-IR categories (from the 1st to 4th quartile).

Table 3 represents the ORs of MetS according to the HOMA-IR categories. In general, it seems clear that the OR of developing MS increases as a function of the severity in HOMA-IR. Compared with children in the 1st HOMA-IR quartile, children in the 4th HOMA-IR quartile had significantly higher ORs for having abnormalities in WC (*p* < 0.001), SBP (*p* = 0.001), DBP (*p* = 0.001), FBG (*p* < 0.001), TG (*p* < 0.001), TC (*p* < 0.001), and HDL-C (*p* < 0.001). The ORs for children in the 4th HOMA-IR quartile to have abnormalities in SBP (*p* = 0.051), DBP (*p* = 0.005), FBG (*p* < 0.001), TG (*p* < 0.001), TC (*p* = 0.016), and HDL-C (*p* = 0.006) remained significant even after adjustments for age, gender, BMI, and body fat percentage. Similarly, children in the 3rd HOMA-IR quartile had significantly higher ORs of developing abnormalities in WC (*p* < 0.001), FBG (*p* < 0.001), TG (*p* < 0.001), and HDL-C (*p* < 0.001), as compared with children in the 1st HOMA-IR quartile. The ORs for children in the 3rd HOMA-IR quartile to have abnormalities in FBG (*p* < 0.001), TG (*p* = 0.001), and HDL-C (*p* = 0.010) remained significant even after adjustments for age, gender, BMI, and body fat percentage.

**Figure 1:**
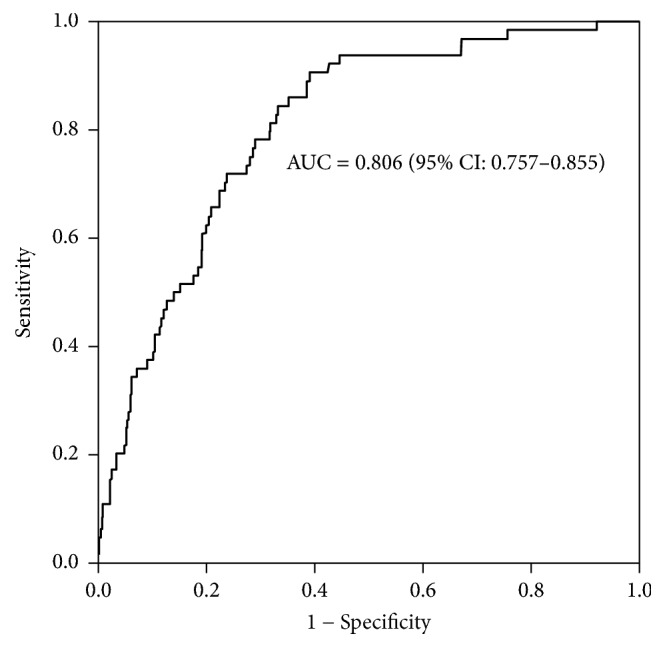
Receiver operating curve analysis of HOMA-IR values for metabolic syndrome in the study population.

Table 4 shows the association between IR and MetS among children in the study. Children in the 4th HOMA-IR quartile had a significantly higher OR (OR = 6.65, 95% CI = 3.89–11.38, *p* < 0.001) of MetS, as compared with children in the 1st HOMA-IR quartile (referent, OR = 1). The risk for children in the 4th HOMA-IR quartile to have MetS remained significant (OR = 3.79, 97% CI = 1.96–7.32, *p* < 0.001) even after adjustments for age, gender, and obesity parameters such as BMI, body fat percentage, WC, and WHR. Furthermore, a receiver operating characteristic (ROC) curve analysis, as illustrated in Figure 1, showed that the area under the curve of HOMA-IR for the diagnosis of MetS was 0.806 (95% CI = 0.757–0.855, *p* < 0.001) in this study population.

## 4. Discussion

This study examined the association between IR and MetS in healthy children aged 7–13 years in Korea. Findings from the current study suggest that a higher IR index assessed by the HOMA-IR value is positively associated with an increase in abnormalities in each component of MetS in the pediatric population. In particular, children in the highest HOMA-IR quartile were at a significantly increased risk for having MetS even after adjustments for the covariates including age, gender, and obesity parameters, as compared with children in the lowest HOMA-IR quartile. Furthermore, the result of ROC curve analysis indicates the diagnostic potential of the HOMA-IR value to identify children with MetS for intervention early in life. This finding is clinically relevant since the link between IR and metabolic disorders associated with MetS in adulthood begins in childhood.

Our findings extend the previous observations on IR in children and adolescents in Korea. In a population-based study, Yi et al. [13] reported that IR assessed by the >95th percentile of HOMA-IR was significantly associated with abnormalities in MetS components in 2716 children and adolescents aged 10–20 years in Korea. Additionally, the risk of IR with both hypertriglyceridemia and hyperglycemia was independent of adiposity in their study population. In another cross-sectional study involving 817 healthy adolescents aged 15-16 years in Korea, Lim et al. [10] reported that subjects in the ≥95th percentile HOMA-IR category had significantly higher risks for developing abnormalities in BP, TC, TG, and liver enzymes even after adjustments for age and gender, as compared with subjects in the <50th percentile HOMA-IR category. Likewise, Juárez-López et al. [12] showed that regardless of age and gender, the severity of IR assessed by the HOMA-IR value was positively associated with the prevalence of abnormalities in each MetS component in obese children aged 10–13 years in Mexico; they further found that the risk of MS was additive according to incremental age- and gender-specific HOMA-IR percentiles (from low to high). Together, those findings suggest that IR may play a critical role in the clustering of MetS risk factors even in apparently healthy children and/or adolescents.

There are several potential explanations for the relationship between IR and the clustering of MetS risk factors. First, IR may be an underlying mechanism leading to dyslipidemia featuring increased TG and decreased HDL-C among MetS components. IR results in an elevation in fatty acid release from adipose tissue and hepatic TG synthesis, along with a subsequent increase in VLDL release into circulation, contributing to elevated plasma TG levels [14]. Hypertriglyceridemia further leads to decreased HDL as a result of enhanced clearance of TG-enriched HDL by hepatic lipase [15].

Second, it is well established that IR is associated with elevated BP in patients with essential hypertension [16]. Data from the Cardiovascular Risk in Young Finns study showed that fasting insulin levels in children predicted their blood pressure 6 years later [17]. Hyperinsulinemia as a compensatory response to IR increases sodium retention and stimulates the sympathetic nervous system [18]. In addition, insulin induces oxidative stress, leading to free-radical damage that impairs the function of endothelial cells [19], and also acts like a growth factor that thickens blood vessels and increases the risk of cardiovascular diseases [14, 20].

Third, obesity may be an important link between IR/hyperinsulinemia and MetS in children and adolescents. We found positive, linear trends in BMI, body fat percentage, and WC according to the incremental HOMA-IR categories in this study population. Likewise, Lim et al. [10] reported that, regardless of gender, HOMA-IR was significantly associated with obesity parameters including weight, BMI, WC, and WHR in healthy children and adolescents aged 15-16 years in Korea. In a review paper, Chiarelli & Marcovecchio [1] reported that regardless of age, gender, ethnicity, and pubertal stage, total adiposity was the major determinant of IR in childhood and adolescence, which explained approximately 55% of the variance in insulin sensitivity in children [21]. Obese children have hyperinsulinemia and significantly lower insulin-stimulated glucose disposal than nonobese children [22]. Furthermore, the rise in childhood obesity is closely related to the rise in the incidence of T2D in the pediatric population [23].

Additionally, we found that girls had significantly higher body fat percentage (*p* = 0.007) and fasting FBG (*p* = 0.005) values than boys in this study population. Furthermore, girls tended to have a higher prevalence of MetS (*p* = 0.051) than boys. Together, these findings suggest that, in childhood, girls are at an increased risk of IR and thereby MetS than boys in Korea. In an agreement with this notion, Yi et al. [13] found that females had a peak HOMA-IR value at 12-13 years of age, while males had a peak HOMA-IR value at 14-15 year of age. Juárez-López et al. [12] compared IR markers between children and adolescents aged 11–13 years in Mexico and found that girls had significantly higher insulin and HOMA-IR values and lower FBG value than boys, implying hyperinsulinemia as a compensatory mechanism to maintain normal FBG levels. Consequently, gender differences in adiposity, pubertal stage, growth, or sexual hormones may explain the gender differences in IR and its risk factors between boys and girls [24], which should be investigated in a future study.

This study has several limitations. First, the cross-sectional nature of this population- based study does not allow for any causal inferences regarding IR and its relationship with abnormalities in MetS components. Second, lifestyle factors such as caloric intakes, physical activity, and TV or computer screen time, which might influence the association between IR and MetS, were not available in this study. Third, data on pubertal stage, growth, and sexual hormones, which might also influence the association between IR and MetS, were not available in this study. Fourth, systemic low-grade inflammation is a characteristic of the IR state. In obese subjects, adipose tissue synthesizes and secretes higher levels of inflammatory cytokines such as TNF-*α* and IL-6, increasing hepatic C-reaction protein (CRP). Obesity is associated with elevated CRP levels in children [25, 26]. Therefore, a further study will be necessary to investigate the role of inflammatory cytokines as a link between IR and MS in healthy children. Lastly, several definitions of MetS are available, with some different outcomes depending on which one is used. Consequently, a further study will be necessary to study how the definition of MetS influences the association between IR and MetS risk factors in children.

In summary, this study investigated the association between IR and MetS in a sample of healthy children in Korea and found that the severity of IR is positively associated with the clustering of MetS risk factors in healthy Korean children, implying a diagnostic tool of the IR index that identifies children at increased risk of IR to prevent metabolic disorders secondary to IR in young adulthood.

## 5. Conclusion

IR is a hallmark of obesity, diabetes, and cardiovascular diseases and leads to several abnormalities in the components of MetS [1]. IR develops during childhood and adolescence and can progress to T2D and CVD during young adulthood, contributing to increased morbidity and mortality. Thus, clinical and public health implications of the current findings are evident. An intervention specifically targeting the insulin-sensitizing effect of physical activity may confer some benefits beyond growth and development, justifying the need to promote physical activity and fitness for metabolic and cardiovascular health even in childhood.

## Figures and Tables

**Table 1:** Description of anthropometrics and metabolic risk factors in study participants.

Variables	Total (*n* = 1036)	Boys (*n* = 488)	Girls (*n* = 548)	*p* value
*Anthropometrics*				
Age (years)	10.5 ± 1.7	10.1 ± 1.7	10.8 ± 1.7	0.198
BMI (kg/m^2^)	19.1 ± 3.4	19.1 ± 3.3	19.3 ± 3.4	0.409
Body fat (%)	11.1 ± 7.8	10.3 ± 6.4	11.7 ± 8.8	0.007
WC (cm)	67.7 ± 11.3	67.1 ± 11.3	68.3 ± 11.2	0.091
*Blood pressure*				
SBP (mmHg)	107.1 ± 14.5	107.3 ± 15.3	106.9 ± 13.8	0.683
DBP (mmHg)	65.2 ± 10.5	64.8 ± 10.5	65.5 ± 10.4	0.288
*Metabolic risk factors*				
TG (mg/dL)	88.4 ± 47.9	86.8 ± 48.1	89.7 ± 47.7	0.336
TC (mg/dL)	174.0 ± 32.3	173.7 ± 32.7	174.1 ± 32.0	0.841
HDL-C (mg/dL)	56.0 ± 13.4	56.3 ± 13.5	55.7 ± 13.2	0.476
Glucose (mg/dL)	91.1 ± 9.7	90.3 ± 8.7	91.9 ± 10.5	0.005
Insulin (*μ*U/mL)	9.1 ± 6.0	9.0 ± 5.7	9.2 ± 6.2	0.530
HOMA-IR	2.1 ± 1.4	2.0 ± 1.4	2.1 ± 1.5	0.336
*Metabolic syndrome (*%)				
0	48.4	55.7	41.8	0.055
1	31.7	29.3	33.8	0.020
2	14.3	10.5	17.7	<0.001
≥3 components	5.7	4.5	6.8	0.051

BMI: body mass index; WC: waist circumference; SBP: systolic blood pressure; DBP: diastolic blood pressure; TG: triglycerides; TC: total cholesterol; HDL-C: high density lipoprotein-cholesterol.

**Table 2:** Anthropometrics and metabolic risk factors according to HOMA-IR quartiles.

Variables	HOMA-IR quartiles				
	Q1	Q2	Q3	Q4	*p* for linear
	(<1.13)	(1.13–1.65)	(1.65–2.65)	(>2.65)	trend
*Age (years)*	10.3 ± 1.9	10.3 ± 1.8	10.8 ± 1.6	11.4 ± 1.3	<0.001
*Body composition*					
BMI (kg/m^2^)	17.3 ± 2.6	18.4 ± 2.8	19.6 ± 3.2	21.4 ± 3.6	<0.001
Body fat (%)	7.9 ± 6.5	8.6 ± 6.4	10.1 ± 6.4	16.5 ± 8.2	<0.001
WC (cm)	62.5 ± 10.2	64.9 ± 10.3	69.2 ± 10.6	74.5 ± 10.1	<0.001
*Blood pressure*					
SBP (mmHg)	104.2 ± 14.8	106.1 ± 13.0	107.4 ± 13.7	110.8 ± 15.2	<0.001
DBP (mmHg)	63.8 ± 11.0	64.1 ± 8.8	65.1 ± 8.9	66.7 ± 11.8	<0.001
*Metabolic profiles*					
TG (mg/dL)	70.0 ± 29.9	77.9 ± 41.1	95.5 ± 50.1	110.8 ± 55.2	<0.001
TC (mg/dL)	169.3 ± 29.5	171.3 ± 29.5	173.3 ± 27.8	182.7 ± 39.1	<0.001
HDL-C (mg/dL)	61.3 ± 13.5	58.7 ± 12.6	53.8 ± 12.8	50.3 ± 11.9	<0.001
Glucose (mg/dL)	86.2 ± 8.2	90.9 ± 8.6	91.6 ± 8.1	95.5 ± 10.7	<0.001
Insulin (*μ*U/mL)	3.8 ± 1.1	6.2 ± 0.9	9.3 ± 1.5	17.0 ± 6.3	<0.001

BMI: body mass index; WC: waist circumference; SBP: systolic blood pressure; DBP: diastolic blood pressure; TG: triglycerides; TC: total cholesterol; HDL-C: high density lipoprotein-cholesterol; FBG: fasting blood glucose. *p* values were adjusted for age and sex using general linear models.

**Table 3:** Odds ratio (OR) and 95 confidence interval (95% CI) of metabolic abnormalities according to HOMA-IR quartiles.

Metabolic abnormalities	HOMA-IR quartiles (values^*∗*^)			
	Q1	Q2	Q3	Q4
	(<1.13)	(1.13–1.65)	(1.65–2.65)	(>2.65)
*WC ≥ 90 percentile*				
Unadjusted OR	Referent	1.48	3.89	8.40
95% CI	-	0.78–2.78	2.22–6.80	4.89–14.42
*p* value	-	0.225	<0.001	<0.001
Adjusted OR	Referent	0.58	1.10	1.68
95% CI	-	0.37–0.89	0.73–1.63	1.12–2.50
*p* value	-	0.189	0.813	0.182
*SBP ≥ 90 percentile*				
Unadjusted OR	Referent	1.36	1.70	2.41
95% CI	-	0.75–2.43	0.96–2.97	1.40–4.12
*p* value	-	0.303	0.065	0.001
Adjusted OR	Referent	1.14	1.34	1.90
95% CI	-	0.83–1.57	0.97–1.85	1.35–2.66
*p* value	-	0.662	0.344	0.051
*DBP ≥ 90 percentile*				
Unadjusted OR	Referent	1.00	1.33	1.99
95% CI	-	0.63–1.57	0.86–2.04	1.31–3.01
*p* value	-	1.000	0.198	0.001
Adjusted OR	Referent	0.91	1.25	2.03
95% CI	-	0.71–1.16	0.97–1.59	1.56–2.63
*p* value	-	0.687	0.349	0.005
*Glucose ≥ 100 mg/dL*				
Unadjusted OR	Referent	4.15	5.63	15.62
95% CI	-	1.76–9.73	2.45–12. 92	7.04–34.63
*p* value	-	0.001	<0.001	<0.001
Adjusted OR	Referent	4.97	6.70	19.54
95% CI	-	3.07–8.03	4.32–11.1	12.1–31.3
*p* value	-	0.001	<0.001	<0.001
*TG ≥ 150 mg/dL*				
Unadjusted OR	Referent	2.50	7.43	15.11
95% CI	-	0.88–7.33	2.85–19.34	5.95–38.35
*p* value	-	0.083	<0.001	<0.001
Adjusted OR	Referent	1.81	4.56	7.64
95% CI	-	1.06–3.06	2.82–7.34	4.73–12.34
*p* value	-	0.246	0.001	<0.001
*TC ≥ 200 mg/dL*				
Unadjusted OR	Referent	0.94	1.13	2.34
95% CI	-	0.58–1.54	0.70–1.80	1.45–3.45
*p* value	-	0.817	0.618	<0.001
Adjusted OR	Referent	0.92	1.06	1.83
95% CI	-	0.70–1.20	0.81–1.37	1.41–2.38
*p* value	-	0.748	0.820	0.016
*HDL-C < 40 mg/dL*				
Unadjusted OR	Referent	1.09	3.33	5.39
95% CI	-	0.71–1.67	2.333–4.77	3.81–7.61
*p* value	-	0.830	<0.001	<0.001
Adjusted OR	Referent	1.00	2.52	2.70
95% CI	-	0.64–1.54	1.74–3.64	1.86–3.91
*p* value	-	0.999	0.010	0.006

WC: waist circumference; SBP: systolic blood pressure; DBP: diastolic blood pressure; TG: triglycerides; HDL-C: high density lipoprotein-cholesterol. ^*∗*^Adjusted for age and sex. Adjusted OR for age, sex, BMI, and body fat percentage.

**Table 4:** Odds ratio (OR) and 95% confidence interval (95% CI) of the 4th HOMA-IR quartile for having metabolic syndrome.

HOMA-IR quartile (value^*∗*^)	OR	95% CI	*p* value
Referent (HOMA-IR of ≥2.65)	1	-	-
Unadjusted OR	6.65	3.89–11.38	<0.001
Adjusted OR	3.79	1.96–7.32	<0.001

HOMA-IR: homeostasis model assessment of insulin resistance. ^*∗*^Adjusted for age and sex. Adjusted OR for age, sex, BMI, body fat percentage, waist circumference, and WHR.
